# Wheat Seed Coating with *Streptomyces* sp. Strain DEF39 Spores Protects against *Fusarium* Head Blight

**DOI:** 10.3390/microorganisms10081536

**Published:** 2022-07-29

**Authors:** Valerio Mattei, Andrea Motta, Marco Saracchi, Andrea Kunova, Paolo Cortesi, Cristina Pizzatti, Matias Pasquali

**Affiliations:** Department of Food, Environmental and Nutritional Sciences, University of Milan, 20133 Milan, Italy; valerio.mattei@studenti.unimi.it (V.M.); andrea.motta6@studenti.unimi.it (A.M.); marco.saracchi@unimi.it (M.S.); andrea.kunova@unimi.it (A.K.); paolo.cortesi@unimi.it (P.C.); cristina.pizzatti@unimi.it (C.P.)

**Keywords:** biocontrol, seed treatment, actinomycetes, *Fusarium graminearum*, strain-specific primers, endophytes

## Abstract

Streptomycetes are promising candidates for the biological control of *Fusarium* Head Blight (FHB) in wheat. Studies involving the use of streptomycetes as biological control agents (BCAs) have been limited to the application when the wheat plant is developed, close to the infection on the spike during flowering. Here, we tested the effects of seed treatment with the *Streptomyces* sp. DEF39 spores before sowing on FHB symptoms’ development. The seed treatment protected the plant from infection by *Fusarium graminearum* by 49% (*p* = 0.04). We traced *Streptomyces* sp. DEF39 in plant organs using strain-specific primers here developed, showing that the streptomycete acts as an endophyte, colonizing the plant tissues up to the spike as well as the roots. This work suggests that it is possible to use a streptomycete as a seed coating BCA, able to partially protect wheat from FHB disease.

## 1. Introduction

Wheat is one of the most important cereals worldwide. However, the development of wheat diseases caused by a group of *Fusarium* spp. causes significant yield and economic losses.

*Fusarium graminearum* is the principal cause of FHB worldwide [1], and it has a high impact on production due to the accumulation of mycotoxins belonging to the trichothecenes family, such as deoxynivalenol (DON), nivalenol (NIV), and their acetylated derivatives, which affect human and animal health [2].

Biological control agents (BCAs) can be exploited alone or in conjunction with a comprehensive FHB disease management strategy [3] to reduce the chemical load on the environment. Indeed, bacteria and fungi are among the biocontrol agents (BCAs) that have been identified and tested in vitro, in the greenhouse, and in the field to compete against *Fusarium* spp. Several mechanisms of action have been described including antibiosis, parasitism, nutrient competition against pathogens, and plant defense triggering.

Among bacterial BCAs, several strains from different species have been successfully tested, such as *Bacillus* spp. [3,4] *Lysobacter enzymogenes* [5], *Pseudomonas* spp. [6,7], and *Streptomyces* spp. [3,8,9,10,11].

Furthermore, different authors observed that *Bacillus* and *Pseudomonas* can prevent *Fusarium* crown rot infections in wheat when inoculated at the seed level [7,8,9,10,11].

Given the endophytic ability of streptomycetes [12,13], we investigated the role of a *Streptomyces* strain, DEF39, as a potential BCA against FHB by applying the inoculum at the seed level. *Streptomyces* sp. DEF39, an endophyte of *Secale cereale,* showed the ability to limit *F. graminearum* growth in vitro [14]. Interestingly when applied to autoclaved wheat seeds, a favorable condition for the high level of DON production, *Streptomyces* sp. DEF39 was very efficient in blocking toxin synthesis by *F. graminearum* [15]. We monitored the distribution of the strain within the plant by a strain-specific PCR, and we observed the level of protection provided, showing that a seed pre-treatment with streptomycete spores upon sowing can effectively decrease the impact of the disease on the spike.

## 2. Materials and Methods

### 2.1. Inoculum Preparation and Treatment

Fungal spore suspensions of *F. graminearum* strain PH-1, provided by Corby Kistler, USDA St. Paul, MN, USA, were prepared in a Carboxy Methyl Cellulose medium(CMC) [14]. The spore suspension was filtered through one layer of gauze and spores were counted and washed in sterile water.

*Streptomyces* sp. DEF39 spores were collected with 10 mL of distilled sterile water by thoroughly scraping the surface of three-week-old cultures, grown on Czapek’s with yeast agar (CZY) plates at 25 °C, with a sterile plastic spatula [14]. The concentration of the spores was brought to 1 × 10^7^ spores/mL and used to soak surface-sterilized wheat seeds of the *Triticum aestivum* ‘Bandera’ [14]. Seed treatment occurred by leaving the inoculated seeds overnight with 3 mL of *Streptomyces* sp. DEF39 spore suspension (10^7^ spores/mL) and drying them under a laminar flow hood. Further, control seeds were treated with 3 mL of deionized sterile water.

Wheat plants were grown in a greenhouse equipped with supplementary light and a cooling system, with a 16-h light (∼PPFD of 600 μmol of photons/(m^2^s^−1^)) and an 8-h dark photoperiod [16]. During the experiment, the recorded average temperatures during the light and dark periods were 24 °C and 18.6 °C, respectively. The relative humidity (HR) percentage averaged between 74.3% and 51.7% during the dark and light periods, respectively. *Triticum aestivum* ‘Bandera’, which is middle-high and susceptible to fungal pathogens, was grown in the greenhouse in pots of 17.5 cm × 19.5 cm, sowing three grains per pot. Plants were grown in a blend (1:1 ratio) of Irish and enriched-black peat-based growth substrate (SER CA-V7 and SER V10-14P, Vigorplant, Italy). Pots were randomly distributed and watered every two days with tap water, maintaining soil moisture at the level of 80% of the full soil water capacity until the plant’s full vegetative growth was reached. During anthesis, spikes were inoculated with *F. graminearum* by spraying 100 µL of spore suspension (10^7^ spores/mL) on the spike (N = 36), or others were inoculated according to Breakspear et al. [17] by cutting the fifth spikelet and inoculating 10 µL of spore suspension (N = 30 spikes). Every single spike was covered with transparent polyethylene plastic bag to maintain humidity for 48 h. Disease assessment was carried out after *F. graminearum* inoculation using a visual scoring system for severity modified from [18] which estimates disease progression expressed on a scale from 0 to 10, each value representing an increase of 10% in the percentage of blight head (0: no infection, 10: 100% of the head blight). The disease was monitored weekly. The final assessment was performed at 28 dpi. Controls with only water and only PH-1 treatment without DEF39 treatment were included in both infection methods.

Nine plants were sampled to re-isolate streptomycetes and other bacteria using CZY and LB medium from the inner root tissues, stems, and leaves.

### 2.2. Design of Strain-Specific Primers

To design strain-specific primers, we retrieved all non-coding sequences of the DEF39 genome (unpublished) using the Microscope platform [19]. Using BlastN, sequences were screened for uniqueness on the nr database. A putative unique non-coding region of 441nt of *Streptomyces* sp. DEF39 genome was identified and selected as a target (Supplementary Figure S1). Subsequently, specific primers for PCR were designed using Primer-blast in the NCBI database, accessed on 10 January 2022. Primer forward (5′-AGTCCGAGGAAGGAACAACG-3′) is at position 65–84, while primer reverse (5′-CCAGCACTGAGAAGCCTCAT-3′) starts at 156nt and stops at 137nt. Using Primer-BLAST online with default settings, primers were generated in a region of the sequence that had no similarity to the NCBI database sequences (accessed the 20 November 2021). Primers were also checked on the nr database, and they were selected if at least two SNPs against the whole nr database could be retrieved.

Two-step PCR was carried out to minimize primer dimers. PCR was performed in a total volume of 25 μL, which contained 0.2 μL of GoTaq^R^ DNA Polymerase 5 U/mL (Promega, Madison, WI, USA), 5 μL of Green GoTaq^R^ Reaction Buffer 5X (Promega, Madison, WI, USA), 2.5 μL of 10 mM dNTP (Promega, Madison, WI, USA), 1 μL of 10 mM primer forward, 1 μL of 10 mM primer reverse, 2 μL of template DNA, and nuclease-free water. The PCR conditions were as follows: 95 °C for 2.5 min, followed by 95 °C for 20 s, 59 °C for 30 s conducting 30 cycles, and then final extension at 72 °C for 5 min. Additionally, PCR using 16S primers (FWD sequence 5′-AGAGTTTGATCCTGGCTCAG-3′; REV sequence 5′-CTACGGCTACCTTGTTACGA-3′) was carried out as positive control for the amplification. PCR was performed in a total volume of 25 µL, which contained 0.2 µL of GoTaq^R^ DNA Polymerase 5 U/mL (Promega, Madison, WI, USA), 5 µL of Green GoTaq^R^ Reaction Buffer 5X (Promega, Madison, WI, USA), 2.5 µL of 10 mM dNTP (Promega, Madison, WI, USA), 2 µL of 10 mM primer forward, 2 µL of 10 mM primer reverse, 1 µL of template DNA, and nuclease-free water. The reaction conditions were initial denaturation at 94 °C for 3 min, followed by 28 cycles of denaturation at 94 °C for 15 s, annealing at 57 °C for 20 s, and extension at 72 °C for 50 s. A final extension was performed at 72 °C for 5 min.

A two-step PCR was also carried out on the DEF39 pure genomic DNA (100 ng/µL) to assess primer sensitivity, with a total of six 1:10 dilutions.

All reaction products were separated by electrophoresis on a 1.5–3% agarose gel containing ethidium bromide and visualized under UV light.

### 2.3. Primers Specificity and In Vivo Tests

DEF39 primer specificity was tested on different *Streptomyces* spp. strains using DNA extracts obtained by [14] (Table 1A), as well as analysing direct PCR results on bacterial colonies obtained from roots isolation on CZY plates using 15 µL DMSO (Amresco, Solon, OH, USA) as a solvent for colony direct PCR procedure [20] (Table 1B). Moreover, wheat spikes obtained from plants treated and not treated at the seed level before sowing were used as DNA sources, obtained according to the DNAeasy Plant Pro Kit protocol (Qiagen, Hilden, Germany) (Table 1C).

### 2.4. Statistical Analyses

All data were organized and analyzed in Microsoft Excel 2021 (Microsoft, Redmond, WA, USA). SuperPlotsOfData [21] from an Excel file was used to represent the results in graphical form.

JASP Stats tool [22] was used for one-way ANOVA with the Mann–Whitney U test (Honestly Significant Difference) (*p* < 0.05).

## 3. Results

### 3.1. Streptomyces *sp.* DEF39 as a Biocontrol Agent

The first objective of the work was to assess the effect of DEF39 as a protectant of the plant against *Fusarium* Head Blight when applied as a seed treatment.

To analyze the effects, we tested two different infection approaches: the infection that injected *F. graminearum* conidia in the spikelet, and the spray inoculation on the spike surface. Interestingly we observed that the average disease level decreased in both types of infection after DEF39 bacterization. With spikelet inoculation, no significant protection could be reached as, on average, the infection was 10% lower but with a *p*-value = 0.55 (Supplementary Figure S2).

**Figure 1:**
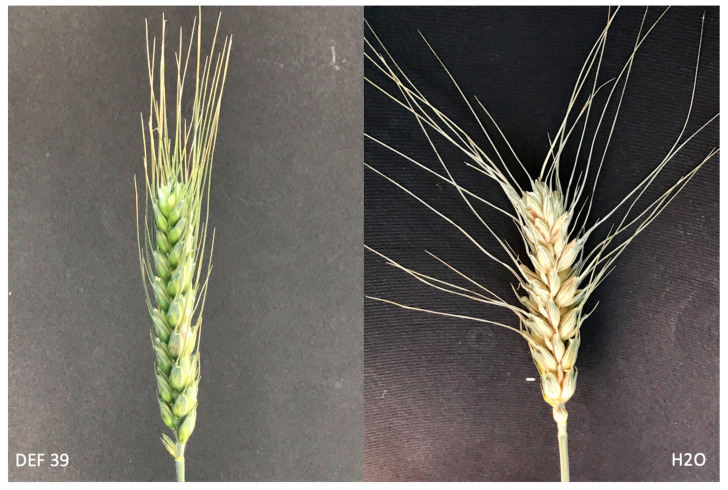
*Fusarium graminearum* spray inoculated spikes. Comparison between DEF 39 seed inoculated plant (**left**) and non-treated plant (**right**) at 21 days from fungal inoculation.

Interestingly, the spray inoculation approach that mimics natural infection led to significant protection (*p* = 0.043) of the plants bacterized by DEF39 (Figure 1).

**Figure 2:**
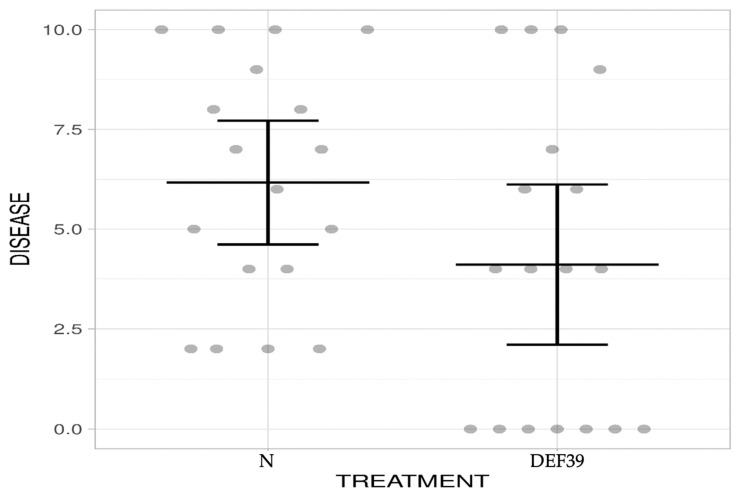
FHB disease severity of wheat bacterized and not with DEF39: Disease assessment was conducted four weeks after spraying *Fusarium graminearum* conidia onto the spikes (*p*-value < 0.043, Mann–Whitney U test).

Our experiment showed a reduction of 49% of the disease severity in the DEF39-treated plants compared to controls (Figure 2). Overall, these data suggest that *Streptomyces* sp. DEF39 treatment at the seed level could be a promising strategy for biocontrol when fungal spores are sprayed, mimicking the natural infection mechanism.

### 3.2. Streptomyces *sp.* DEF39 Specific Primer Validation and Systemic Colonization of the Plant

In order to understand the behavior of DEF39 in wheat plants, we developed a specific PCR to detect DEF39 in different tissues of the plant including the spike.

Primer specificity was tested on different *Streptomyces* spp. genomes and on DNA from bacterial wheat endophytes (Table 1A). PCR experiments demonstrated that primers were able to discriminate *Streptomyces* sp. DEF39 from different *Streptomyces* spp. strains [23], as well as from other bacterial species. In silico testing on available genomes from public databases confirmed the uniqueness of the sequence used for primer design (Supplementary Figure S1). Furthermore, primer sensitivity was tested using serial dilutions of *Streptomyces* sp. DEF39 DNA (100 ng/µL) as a standard solution. Results suggested that the lower detection limit was 0.01 ng/µL (Supplementary Figure S3), corresponding to 22 spores based on copy number per genome size, as described by Pasquali et al. in [24].

Subsequently, the ability of *Streptomyces* sp. DEF39 to translocate within the wheat plant, after seed treatment, was assessed. Indeed, specific primers confirmed the presence of *Streptomyces* sp. DEF39 in different mature plant tissues, i.e., roots and spikes (Supplementary Figure S4).

*Streptomyces* sp. DEF39 was found in all spikes and roots of plants developed from seeds treated with this strain. Results obtained from PCR demonstrated that specific primers detected *Streptomyces* sp. DEF39’s presence in fully matured plants that were previously inoculated at the seed level. Moreover, they also suggested that *Streptomyces* sp. DEF39 can translocate inside wheat plants from seed to both roots and spikes, colonizing the plant in a systemic way.

## 4. Discussion

Our study shows for the first time that the application of seed treatment with spores of an endophytic streptomycete can have effects as a protectant against *Fusarium* Head Blight at the spike. By designing highly-sensitive specific primers, we could confirm that the strain survives effectively in controlled conditions and can colonize the whole plant during its growth. *Streptomyces* sp. DEF39 can colonize the plant, translocating from seeds to both roots and the aboveground tissues up to the spikes.

Interestingly, the presence of the streptomycete has a different effect on the disease depending on the inoculation method of the pathogen. While fungal spray inoculation, which mimics a natural infection, is effectively limited by the presence of DEF39 in the plant, when the infection is induced by cutting the spikelet and introducing directly the fungus into the plant tissue, the effect of DEF39 is limited and does not significantly limit the disease. This can be due to the very harsh infection conditions of the direct inoculation method in the spikelet used in our study. Indeed, it has been observed that different inoculation methods may determine different levels of disease and inform on the type of mechanisms involved in plant resistance to FHB [25].

The inability of DEF39 to protect the spike when conidia are directly introduced in the spikelet suggests that DEF39 may protect spray-inoculated spikes by interacting and modulating the initial plant resistance determining a stop of the pathogen penetration. DEF39 may therefore increase the ability of the plant to limit pathogen penetration, the so-called type 1 resistance, as described by Schroeder and Christensen [26]. Indeed, type 1 resistance is associated with different phytohormone-related pathways that are known to be involved in hormonal crosstalk of any plant–microbe interaction [27], as well as in the expression of a basal defense response [28] that can be triggered by the physical presence or the production by some DEF39 metabolites.

This would be in line with what we observed previously in “micro-silage” conditions [15]. *Streptomyces* sp. DEF39’s efficacy in reducing fungal growth was limited, suggesting that the effects against *F. graminearum* are at least partially mediated by the plant. Further studies can focus on the complex mechanism of protection that DEF39 exploits while interacting with wheat and *F. graminearum*. The ability of *Streptomyces* species to synthesize plant-protective molecules including enzymes, secondary metabolites, and volatile organic compounds, as well as their ability to induce the plant immune system to respond quickly to infections, is what makes them potentially valuable biocontrol agents [9,29,30]. Moreover, streptomycetes have the advantage of not only being a potentially co-evolving force capable of engaging in an arms race with pathogenic species, but many also encode numerous putative antimicrobial biosynthetic gene clusters (BGCs), resulting in the simultaneous production of a plethora of different antibiotics with various modes of action [31]. This could help to slow the rate of resistance evolution. Previous studies in our laboratory [15,18] showed that DEF39 can effectively limit toxin production (DON) without blocking completely fungal growth. This likely occurs via specific metabolites. It is, therefore, possible that the strain also produces metabolites able to modulate the plant response when facing the fungus. This phenomenon has already been demonstrated in the interaction of streptomycetes on maize infected by *Fusarium verticillioides* [32]. The complexity of the interactions occurring between streptomycetes and wheat plant is likely multivariate [30].

To the best of our knowledge, this is the first study in which the ability of a streptomycete as a BCA against *F. graminearum* was assessed after seed inoculum in greenhouse conditions. Previous works on *Streptomyces* spp. applied as seed coatings proved successful on soil diseases such as *Verticillium* Wilt on cotton [33] or *Rhizoctonia* damping-off in tomato [34]. Our work opens up possible novel approaches in the use of endophytes, such as streptomycetes, that can colonize the whole plant and act as protectants when applied as seed inoculum. Positive traits such as the resistance of *Streptomyces* spores to environmental stresses can lead to the development of novel strategies for the organic control and precision agriculture of important plant diseases such as FHB, limiting the interventions in the field [35]. Field studies to verify the fitness and efficacy of the strain in farming conditions as well as genomic studies to decipher the potential mode of action and the molecules involved in the bacteria–plant interaction [36] are warranted.

## Figures and Tables

**Table 1:** Samples used to test *Streptomyces* sp. DEF39 primers specificity. (**A**) Pure DNA extract of strains of our collection. *: Strains identified in [23]; (**B**) Bacterial colonies re-isolated on plates from wheat plants treated with DEF39; (**C**) Bacterial DNA extracted from wheat spikes using DNAeasy Plant Pro Kit. PCR on the 16S region was carried out to confirm negative results obtained from the *Streptomyces* sp. DEF39 specific primer pair. Plants which resulted negative to specific primers were not inoculated with *Streptomyces* sp. DEF39 and were used as a negative control. On the other hand, positive results were obtained in plants previously inoculated at the seed level.

A				
*Streptomyces* spp. Strain Code	Source of Isolation	Positive To DEF39 Specific Primers	Positive To 16S Primers	Genbank Accession Number
DEF07 *	*Camellia japonica*	*-*	*+*	MK412001
DEF09 *	*Triticum aestivum*	*-*	*+*	MK412002
DEF10 *	*Hordeum vulgare* var. *distichum*	*-*	+	MK412003
DEF14 *	*Arundo* sp.	*-*	*+*	MK412005
DEF16 *	*Zea mays*	*-*	*+*	MK412007
DEF17	*Hordeum vulgare*	*-*	*+*	
DEF17B	*Hordeum vulgare*	*-*	*+*	
DEF19 *	*Camellia japonica*	*-*	*+*	MK412008
DEF20 *	*Carex* sp.	*-*	*+*	MK412009
DEF21	*Zea mays*	*-*	*+*	
DEF26	*Triticum aestivum*	*-*	*+*	MK412011
DEF35	*Secale cereale*	*-*	*+*	MK412012
DEF36	*Crocus sativus*	*-*	*+*	
DEF39 *	*Secale cereale*	*+*	*+*	MK412014
DEF47 *	unknown plant	*-*	*+*	MK412018
DEF48 *	*Zea mays*	*-*	*+*	MK412019
**B**				
**Bacteria Re-Isolated on Plates** **from Wheat Plants Treated with DEF39 at the Seed Level**		**Source of Isolation (*Triticum aestivum*)**	**Positive to DEF39 Specific Primers**	**Positive to 16S Primers**
Non-filamentous bacteria		Culm	*-*	*+*
*Streptomyces* sp.		Root	*+*	*+*
*Streptomyces* sp.		Root	*+*	*+*
Non-filamentous bacteria 1		Root	*-*	*+*
*Streptomyces* sp.		Root	*+*	*+*
Non-filamentous bacteria 2		Root	*-*	*+*
Non-filamentous bacteria 3		Root	*-*	*+*
Non-filamentous bacteria 4		Root	*-*	*+*
Non-filamentous bacteria 5		Root	*-*	*+*
Non-filamentous bacteria 6		Seed	*-*	*+*
Non-filamentous bacteria 7		Culm	*-*	*+*
*Streptomyces* sp.		Seed	*+*	*+*
*Streptomyces* sp.		Root	*+*	*+*
**C**				
**Source of Isolation (*Triticum aestivum*)**		**Treatment with DEF 39 at Seed Level**	**Positive to DEF39. Specific Primers**	**Positive to 16S** **Primers**
Spike		-	*-*	*+*
Spike		+	*+*	*+*
Spike		+	*+*	*+*
Spike		+	*+*	*+*
Spike		+	*+*	*+*
Spike		-	*-*	*+*
Spike		-	*-*	*+*
Spike		+	*+*	*+*

## Data Availability

All data are available in the article and Supplementary Materials.

## Supplementary Materials

The following supporting information can be downloaded at: https://www.mdpi.com/article/10.3390/microorganisms10081536/s1, Figure S1: Unique non-coding region of *Streptomyces* sp. DEF 39 genome (441nt). Figure S2: Disease severity of plants treated (DEF39) and not treated (H2O) with DEF39 when *F. graminearum* PH-1 strain was inoculated by cutting the 5th spikelet. Figure S3: DEF39 specific primer sensitivity. Figure S4: DEF39 specific primer amplification of spikes and roots of plants whose seed was treated and not treated with DEF 39 spores.
