# Supplementary material for: Wheat Seed Coating with Streptomyces sp. Strain DEF39 Spores Protects against Fusarium Head Blight
Source: Microorganisms. 2022 Jul 29;10(8):1536. doi: 10.3390/microorganisms10081536 (PMC9415289; doi:10.3390/microorganisms10081536)
Supplement: Supplementary file 1 [file microorganisms-10-01536-s001.zip › supplementary_figure_S1.pdf]

Figure S1: Unique non-coding region of *Streptomyces* DEF 39 genome (441nt)

GCCACCATGATCCACCAGCGGTGATGATTTGAAGGCACCCCGGTGGCATCGGCTCGGGTC
GCTGAGTCCGAGGAAGGAACAACGGTTCCGGTATCCCGGTCGTCTTTCGGTCCCGGGCCG
CCAAGTGACCACTCAGATGAGGCTTCTCAGTGCTGGTAGCAAAGCCTTTCAGCCGCGCGC
CACTTCGGTGCCCGGGTGGTAGAAAGGGCCGCGAGCTGAGCGCCACCCCGTACGCGAGGG
CGTGGCTCCAGGCGAGCGATCCGGAGTGCTCGCACTCCCCGAGCAGGTCTACGGAGCGGG
GGGTACACCGTGAACGGAGCGTCCGAACGAACGCGCAGCTACCGGCTCCTGCAGGGCGAA
CGTCTCGCGCTCGAGATACGCGGTGAGCCAGGGGTCCTTGTGAGCACCTCAGCGCCGCCG
CCCCTCCCGGGAACGCCGCCG
