# Supplementary material for: Wheat Seed Coating with Streptomyces sp. Strain DEF39 Spores Protects against Fusarium Head Blight
Source: Microorganisms. 2022 Jul 29;10(8):1536. doi: 10.3390/microorganisms10081536 (PMC9415289; doi:10.3390/microorganisms10081536)
Supplement: Supplementary file 1 [file microorganisms-10-01536-s001.zip › supplementary_figure_S3.pdf]

Figure S3: Specific primer sensitivity

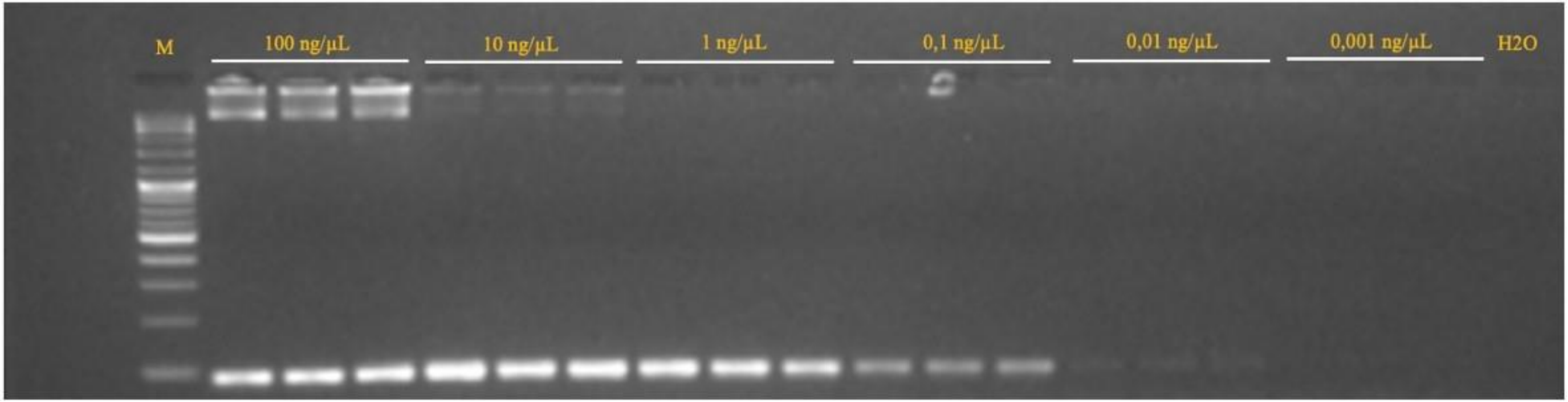

Figure S3: Agarose gel 3% (100 V, 45min) of PCR products obtained using specific primers on different dilutions of Streptomyces DEF 39 genome in triplicate. (100 ng/µL, 10 ng/µL, 1 ng/µL, 0,1 ng/µL, 0,01 ng/µL, 0,001 ng/µL)
