# Supplementary figures and images for: Wheat Seed Coating with Streptomyces sp. Strain DEF39 Spores Protects against Fusarium Head Blight
Source: Microorganisms. 2022 Jul 29;10(8):1536. doi: 10.3390/microorganisms10081536 (PMC9415289; doi:10.3390/microorganisms10081536)

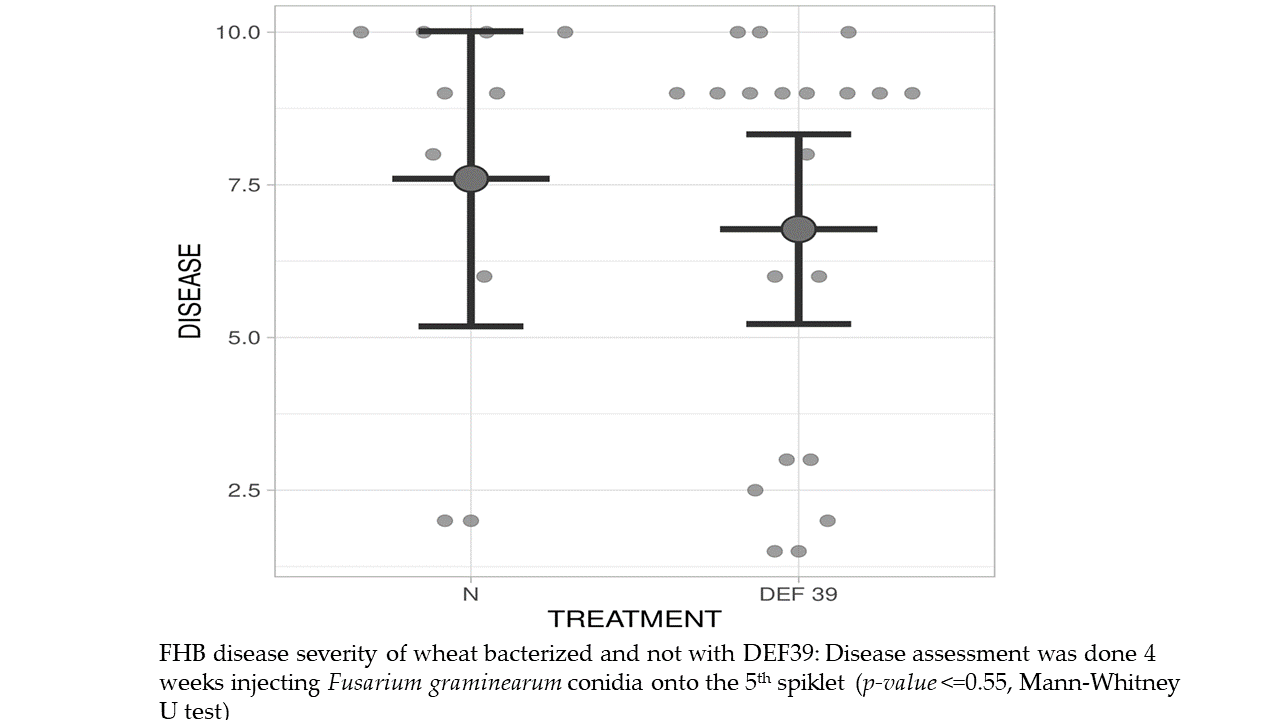

Supplement: Supplementary file 1 [file microorganisms-10-01536-s001.zip › supplementary figure S2.png]

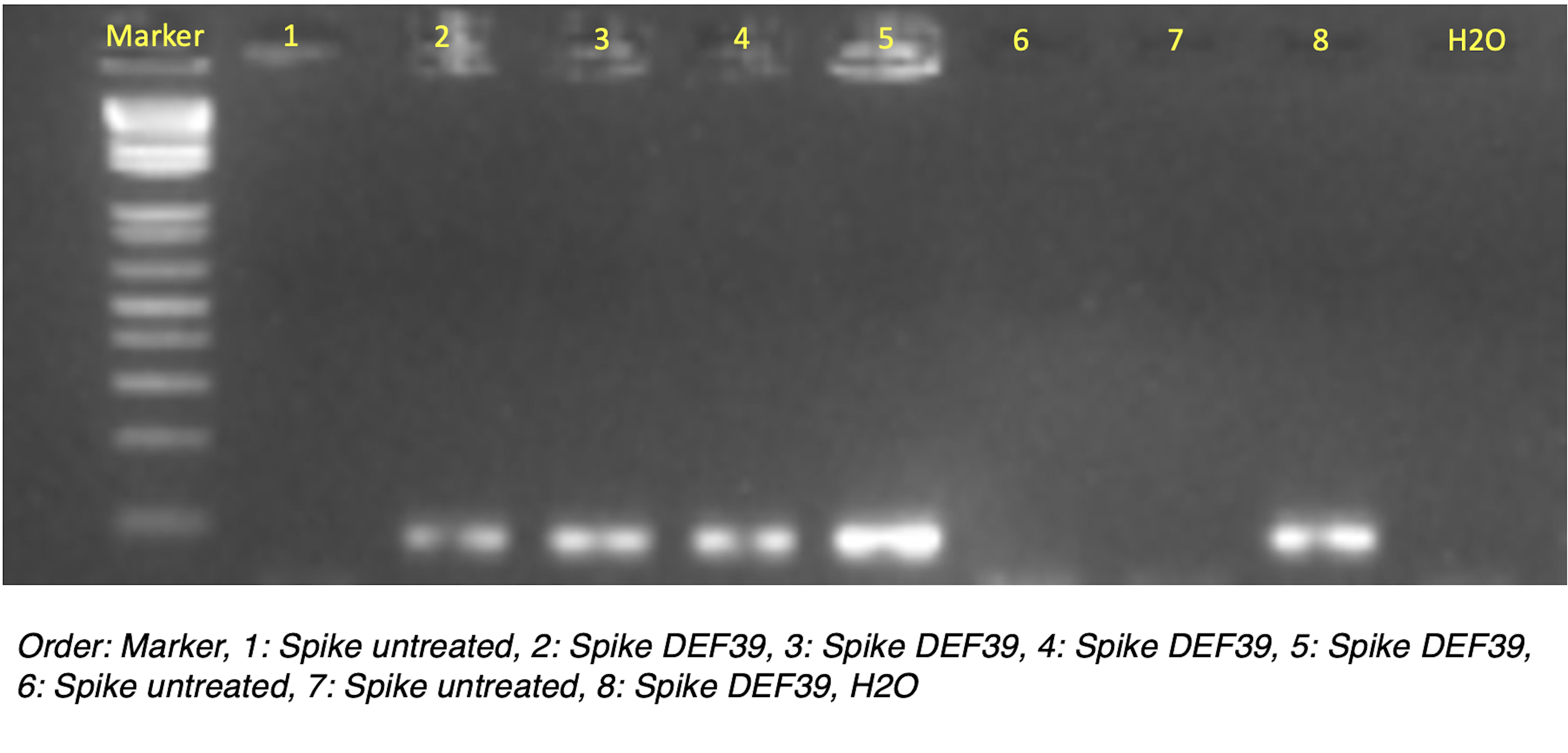

Supplement: Supplementary file 1 [file microorganisms-10-01536-s001.zip › Supplementary figure S4.png]
